# Takotsubo Syndrome: Pathophysiology, Emerging Concepts and Clinical Implications

**DOI:** 10.1161/CIRCULATIONAHA.121.055854

**Published:** 2022-03-28

**Authors:** Trisha Singh, Hilal Khan, David T Gamble, Caroline Scally, David E Newby, Dana Dawson

**Affiliations:** 1 British Heart Foundation Centre for Cardiovascular Science, University of Edinburgh, Edinburgh, United Kingdom; 2 Aberdeen Cardiovascular and Diabetes Centre, University of Aberdeen, Aberdeen, United Kingdom

**Keywords:** Takotsubo Syndrome, Pathophysiology, Outcomes

## Abstract

Takotsubo syndrome is a condition characterized by acute transient left ventricular systolic dysfunction, which at presentation can be challenging to distinguish from acute myocardial infarction. Although previously thought to be a benign, self-limiting condition, recent studies have confirmed that patients with takotsubo syndrome have persistent subtle ongoing cardiac dysfunction, and many continue to have limiting symptoms despite restoration of left ventricular ejection fraction. Moreover, these patients have a substantial burden of morbidity as well as mortality with high rates of subsequent major adverse cardiovascular events that approach those of patients with acute coronary syndrome. The mechanisms behind this condition remain elusive. Despite substantial research, the medical community continues to have an incomplete understanding of its underlying etiology and pathophysiology. Catecholamine-induced myocardial injury is the most established and well-known theory, but this does not explain all of the clinical features and presentations of the condition, and numerous other pathways and abnormalities are emerging. Because of the poor understanding of its underlying pathophysiology, there is a lack of evidence-based interventions to treat the acute episode, to avoid recurrences and to prevent major adverse cardiovascular events. This highlights the need for further research to gain a better understanding of the underlying pathophysiology in order to inform appropriate randomized controlled trials of interventions targeting the causative pathways. Only then can evidence-based management strategies be established to improve clinical outcomes of this potentially lethal condition.

## Introduction

“He first deceased; she for a little tried To live without him; liked it not, and died” -Sir Henry Wootton, 1651

Takotsubo syndrome was first named by Sato et al in 1990,^1^ although sudden and rapid death following intense psychological stress was described by Rees and Engel more than 50 years ago.^2, 3^ Following some initial skepticism, awareness of this potentially fatal condition has substantially increased over the last 10-15 years^4, 5^ and is now an established increasingly frequent and important cause of acute cardiac presentations.^5^

**Figure 1:**
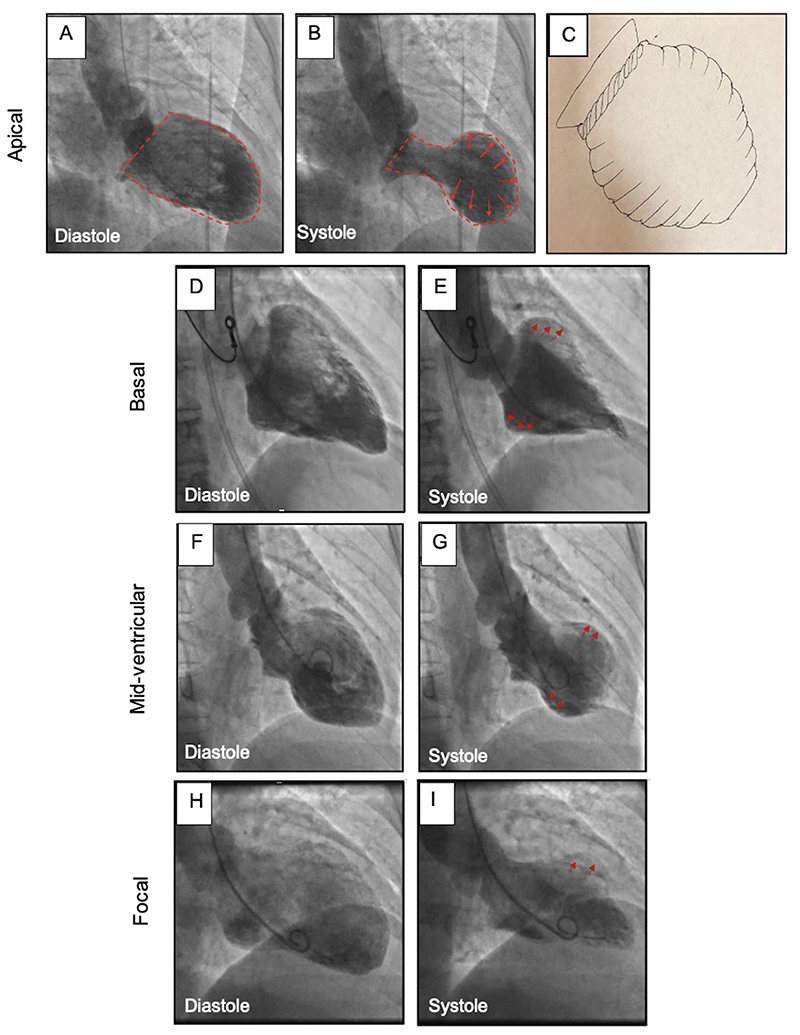
Takotsubo Syndrome: Anatomical variants Left ventriculogram demonstrating apical (A, B) ballooning of the left ventricle, similar to the shape of a Japanese octopus trap (C). Basal (D, E), mid-ventricular (F, G) and focal (H,I) variations of Takotsubo syndrome.

Takotsubo syndrome has several unique characteristics, which distinguish it from other acute cardiac emergencies. It is typified by non-obstructed coronary arteries associated with a characteristic antero-septal-apical dyskinetic “ballooning” of the left ventricle with hyperkinetic basal segments (Figure 1). These features spontaneously resolve to restore *apparently* normal left ventricular function.^6, 7^ Despite being described 30 years ago, its etiology and pathophysiology remains poorly understood and the lack of treatments often presents a clinical dilemma for physicians. Previously believed to be a benign self-limiting condition,^8–13^ takotsubo syndrome is now known to be associated with substantial short and long-term morbidity and mortality. The purpose of this review is to provide an update on the current understanding of the clinical features of this condition, to discuss the latest theories regarding its etiology and pathophysiology, and to highlight the need for future therapeutic interventions to reduce and to prevent the major morbidity and mortality.

## Epidemiology

Takotsubo syndrome is increasing in incidence, which may reflect the rising prevalence of modern life ‘stressors’ as well as the greater awareness and detection of the condition by the clinical cardiology community.^5, 9^ Takotsubo syndrome accounts for approximately 2-3% of all and 5-6% of female patients presenting with acute coronary syndrome,^9, 14, 15^ although it may be under appreciated and under diagnosed especially in patients who have co-existing coronary artery disease.^16^

While the condition can occur in any age group, it characteristically affects women in the sixth decade of life although patients below the age of 50 years account for approximately 10% of cases.^17^ Interestingly, younger patients are more likely to be male, or have ‘atypical’ takotsubo, less co-morbidities, acute neurological or psychiatric disorders and develop in-hospital complications.^17^

Although increasing age may be a risk factor for developing takotsubo syndrome syndrome,^15^ the underlying mechanism remains unknown, but can be partly explained by the influences of age and sex on the sympathetic nervous system. Sympathetic nervous system activity increases with age, particularly in women, and cardiac sympathetic stimulation is augmented due to an imbalance in neuronal norepinephrine homeostasis.^18, 19^ With age, there is a decrease in vagal tone and baroreflex sensitivity with concomitant increase in sympathetic activation,^18^ potentially rendering the myocardium more susceptible to enhanced levels of catecholamines. Although increasing age may be a risk factor for developing takotsubo syndrome, it is not an independent risk factor for in-hospital mortality which contrasts with the well-recognized age-related mortality seen in patients with acute coronary syndrome.^9^

Although most patients (80-90%) who develop takotsubo syndrome are women,^15, 20^ the mechanism underlying this large sex disparity is unknown. Several hypotheses have been proposed and centered on estrogen deprivation occurring post-menopause and its role in regulating sympathetic drive and microvascular blood flow via endothelium-dependent and -independent mechanisms.

Familial takotsubo syndrome has been described^21^ although multigenerational Mendelian inheritance does not occur, suggesting that a genetic predisposition may interact with environmental factors or have a polygenic etiology. Polymorphisms of the beta-1 and beta-2 adrenoreceptors genes have been associated with reduced left ventricular function in patients with takotsubo syndrome, but results are conflicting and inconclusive.^22, 23^ Larger studies are currently underway to explore potential genetic associations (GENETIC- NCT04513054).

## Natural History

Takotsubo syndrome had previously been viewed as an interesting anomaly that ran a benign course. As such, patients were often given reassurances that they were fortunate not to have suffered a heart attack, and that their heart will recover completely back to normal with an excellent prognosis. However, we now know this is not the case. Despite recovery of left ventricular ejection fraction and the absence of major coronary artery disease, patients with takotsubo syndrome have outcomes that are considerably worse than the general population.^8–13^ Takotsubo syndrome has an in-hospital mortality that is comparable to acute ST segment elevation myocardial infarction.^9–11, 13^ Beyond the acute event, patients with takotsubo syndrome have a rate of all-cause death of 5.6% per patient-year and a rate of major adverse cardiac and cerebrovascular events of 9.9% per patient-year.^9^

As many as 1 in 8 patients will experience a repeat acute takotsubo syndrome episode within 5 years of the index event^24, 25^, often precipitated by a further (and often different) stressful event although no known clinical or psychological factors can predict the likelihood of recurrence. Moreover, many patients report substantial morbidity following takotsubo syndrome. Symptoms of dyspnea, lethargy, palpitations and fleeting chest pains can persist for 2 or more years after the index event despite ‘normalization’ of left ventricular ejection fraction. Thus, there is a substantial burden of long-term morbidity and mortality associated with takotsubo syndrome.

## Clinical Features

Patients with takotsubo syndrome classically present with acute onset chest pain, dyspnea and changes on the electrocardiogram occurring in association with an acute stressful event.^15^ This presentation mimics, and is often initially managed as, an acute coronary syndrome (Table 1). Indeed, when marked, it can include presentations consistent with an acute ST segment elevation myocardial infarction. In extreme circumstances, patients can present with severe heart failure, cardiogenic shock or arrhythmias requiring hemodynamic and sometimes ventilatory support.

A precipitating “stressful event” is typically considered to be unexpected bereavement, conflict or major life event. However, it is important to appreciate that a third of patients do not recognize an identifiable stressor, and this is reflected in the latest diagnostic criteria.^15^ This figure may be an over estimate as some patients may be reluctant to discuss personal stressful circumstances, or clinicians may not fully explore or probe underlying stress-related or mental health problems. Furthermore, protracted prior stress may contribute, and this is often undisclosed and under-appreciated. Takotsubo syndrome can also be precipitated following major events resulting in ‘community stress’, such as earthquakes (New Zealand 2010, 2011, and 2016)^26^, and more recently, anxiety related to COVID-19 pandemic.^27^

Non-cardiac medical conditions, particularly neurological, such as intra-cranial hemorrhage, pheochromocytoma and epilepsy, or severe acute critical illnesses can induce takotsubo syndrome.^28–30^ Cancer is increasingly recognized as a precipitant and this may relate to the direct mental stress from receiving a diagnosis of malignancy or the combined mental and physical stress of cancer treatments.^31^ Stresses from a range of physical illnesses or procedures are also recognized and include acute exacerbations of asthma or chronic obstructive pulmonary disease, endoscopic examinations, cardioversions and many others.

Acute or chronic mental health conditions, such as depression and anxiety, are present in a third of patients with takotsubo syndrome.^8, 9, 32^ Rates of psychiatric or neurologic disorders are 7-fold higher than in patients with acute coronary syndrome, with a high prevalence of type-D personality characteristics (negative emotions, social inhibition) suggesting a predisposing element that has not been fully explored by psychiatrists or clinical psychologists.^33^ Patients affected by depression and anxiety also demonstrate overactivity of the sympathetic nervous system in response to physical or emotional stress, implying greater susceptibility to takotsubo syndrome.^33^

The multicenter Spanish REgistry for TAKOtsubo syndrome (RETAKO)^10^ and the International Takotsubo Registry (Inter-TAK)^1, 32^ have shown that patients with an emotional stressor are more likely to be female, present with chest pain and have better outcomes. Interestingly, those with a physical stressor (infection, surgery, trauma, neurological disorder and hypoxia) are more likely to be men, have comorbidities, present with syncope and dyspnea, develop the ‘basal’ pattern of left ventricular dysfunction and have acute complications.^8, 10, 32^

## Investigations

The investigation of takotsubo syndrome may occur either because it is the primary suspected diagnosis or, more commonly, following initial investigation for acute coronary syndrome due to the marked overlap in clinical presentation. A multimodal imaging approach is often needed to discriminate between the differential diagnoses of the acute cardiac presentation (Table 1), with cardiac magnetic resonance playing an increasingly important role.^34^

### Electrocardiogram

The electrocardiogram commonly demonstrates acute dynamic changes at presentation, resembling those of an acute coronary syndrome.^25^ The commonest abnormalities are ST elevation, T wave inversion, and left bundle branch block. ST elevation and T wave inversion are widespread and may not localize to a particular territory.^35, 36^ Three evolving electrocardiographic stages of takotsubo syndrome have been described. Stage 1 involves ST deviation occurring within the first few hours of symptoms onset.^36^ Stage 2 involves progressive deep T wave inversion and QTc prolongation, occurring within 1-3 days and peaking at 2-6 days, although when corrected for gender, the QTc is not different from male patients with acute myocardial infarction.^37^ These T wave inversions are usually widespread over the precordial (V1-6), bipolar (I, II) and lateral limb (aVL) leads and to correlate with myocardial edema, which can persist beyond ventricular contractile recovery.^36^ During these first 24-48 hours, torsade de pointes and other ventricular tachycardias can occur. The association of ventricular arrhythmias with prolonged QTc segment in takotsubo syndrome is well established. However, ventricular arrhythmias in the hyperacute phase of takotsubo syndrome are unlikely to be related to QTc prolongation.^35^ It is also possible that patients present with ventricular arrhythmia and are only subsequently diagnosed with takotsubo syndrome, making it very difficult to understand which occurred first. Stage 3 involves gradual resolution of T wave and QTc changes over subsequent weeks or months.^35^ The normalization of myocardial contractile function occurring prior to long-lasting electrocardiographic abnormalities is in stark contrast with the simultaneous recovery of both contractile function and the electrocardiogram seen after restoration of blood flow in ischemic myocardial stunning. Together with a relative absence of isolated ST-depression on presentation electrocardiogram, these concepts would argue against classical myocardial ischemia playing a part in the etiology of takotsubo syndrome.

Other electrocardiographic findings have also been reported. A J wave and fragmented QRS during the hyper-acute period has been described^38^ as well as low voltage QRS complexes. In those presenting with ST segment elevation, the elevation is more subtle or of lower voltage compared to patients with acute myocardial infarction.^35^ Q waves may also occur acutely and disappear rapidly with re-appearance of the R wave. In conclusion, subtle differences in electrocardiographic changes exist between takotsubo syndrome and acute coronary syndrome. The presence of ST-segment elevation will impact whether the patient undergoes coronary angiography as an emergency or the procedure is slightly deferred.

### Cardiac and Inflammatory Biomarkers

Plasma cardiac troponin concentrations are typically raised although peak values are lower than patients with ST-elevation myocardial infarction and are more comparable to those presenting with non-ST elevation.^39, 40^ Indeed, the rise in cardiac troponin is often disproportionately lower than the associated degree of left ventricular contractile impairment would suggest. This likely reflects the absence of myocardial necrosis, which is more akin to myocardial infarction. In contrast, plasma B-type natriuretic peptide (BNP), and its N-terminal inactive molecule (NT-proBNP), concentrations are typically much higher than those seen in patients with myocardial infarction.^41, 42^ Moreover, NT-proBNP concentrations are particular high in patients with ‘apical’ compared to ‘atypical’ variants, which may reflect the greater degree of acute left ventricular dilatation and myocardial stretch.

Several pro-inflammatory (interleukin-2, interleukin-4, interleukin-8, interferon and tumor necrosis factor alpha) and anti-inflammatory (interleukin-10) cytokines are raised at presentation and can continue to remain elevated for several months thereafter, reflecting the inflammatory pathophysiology of takotsubo syndrome.^43^ Interestingly, interleukin-6 concentrations tend to be higher in patients with acute coronary syndrome, potentially reflecting the greater extent of myocardial necrosis.^44^

### Coronary angiography and left ventriculography

The diagnosis of takotsubo syndrome is often made once an invasive coronary angiogram has been performed and normal or non-obstructive coronary artery disease documented. Co-existing coronary artery disease is present in approximately 15% of patients with takotsubo syndrome^10, 13^ and careful correlation between angiography and the wall motion abnormalities is required. Where doubt exists, advanced intravascular imaging techniques, such as optical coherence tomography and intravascular ultrasound, may help to exclude plaque rupture, which is not a characteristic of takotsubo syndrome.^45^ Other types of myocardial infarction with non-obstructive coronary artery disease or spontaneous coronary artery dissection, also require exclusion based on careful inspection of the coronary angiogram in combination with cardiac magnetic resonance imaging.

Left ventriculography usually confirms the diagnosis due to the characteristic ballooning of the left ventricle. In the majority (50-80%) of cases, there is a typical pattern of apical and mid-ventricular dyskinesis, akinesis or hypokinesis with basal sparing (Figure 1).^15^ The second commonest form affects the mid-ventricle as a circumferential mid-ventricular wall motion abnormality with basal and apical hyperkinesis, this pattern, which is pathognomonic for takotsubo syndrome (Figure 1).^46^ Occasionally, patients may present with a ‘reverse takotsubo syndrome’ where the basal segments are affected with sparing of the mid and apical segments. The incidence of right ventricular involvement can vary (10-30%) and is associated with more severe disease and complications. Finally, other rarer variants have been described, involving isolated right ventricular or focal (segmental) left ventricular takotsubo syndrome.^47^

### Echocardiography

Echocardiography can be used to help support the diagnosis (extent, severity and location of the wall motion abnormalities) and to identify potential complications of takotsubo syndrome. Approximately 20% of patients have evidence of left ventricular outflow tract obstruction.^48^ Usually occurring in those with the typical apical to mid cavity ballooning with basal hyperkinesia and can be associated with mitral regurgitation secondary to systolic anterior motion of the mitral valve apparatus. Ultrasound contrast agents can be useful to delineate wall motion abnormalities and assess for left ventricular thrombus formation, which can occur acutely or later in the disease course.^49^ More advanced techniques, such as speckle-tracking echocardiography, can also show abnormalities of left ventricular twist and lower mean values of systolic peak velocity, strain and strain rate.^50^ Whilst left ventricular twist and deformation indices typically improve during the recovery phase, persistently abnormal cardiac deformation indices suggest a phenotype of heart failure with preserved ejection fraction.^51^

### Cardiac magnetic resonance

**Figure 2:**
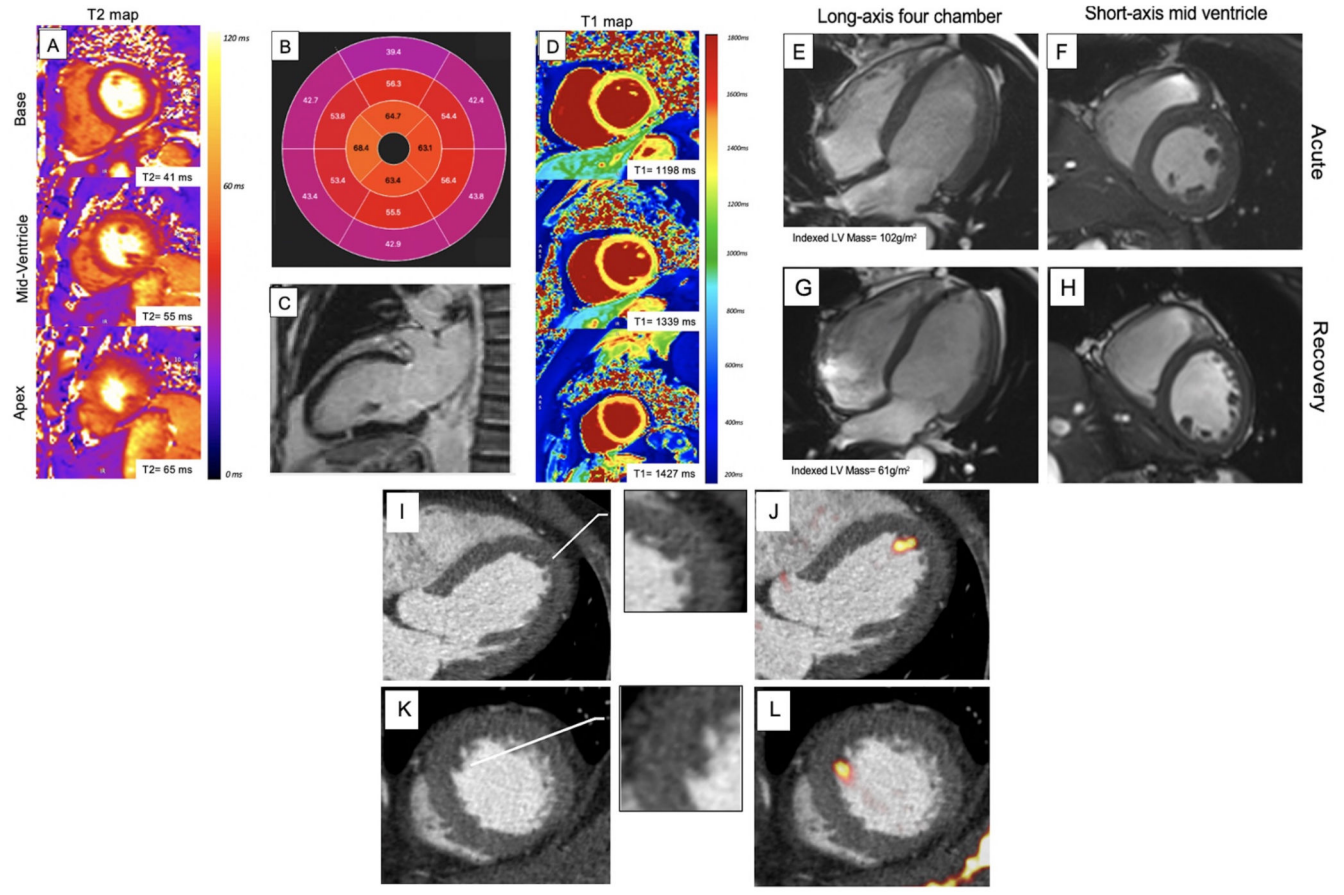
Cardiac Magnetic Resonance Imaging and Computed Tomography Findings in Takotsubo Syndrome Short axis T2 maps (A), T2 polar map (B) and short-axis T1 maps (D), demonstrating elevated T2 and T1 values circumferentially in the mid and apical regions (outside of a coronary territory). Long axis two-chamber view demonstrating transmural fibrotic band pattern typical of takotsubo syndrome 4 months after the index event (C). Long axis four-chamber and short axis views of mid-ventricle demonstrating elevated left ventricular mass in acute phase (E, F) and normalization during convalescence (G, H). Hybrid positive emission tomography with cardiac computed tomography angiography depicting a small left ventricular thrombus in a patient with takotsubo syndrome with no clinically apparent thrombus on conventional imaging. There is subtle hypoattenuation on the computed tomography angiogram (magnified inserts) and increased uptake of an activated platelet and thrombus-specific radiotracer (^18^F-GP-1; yellow-red) in long axis four-chamber (I, J) and short axis (K, L) views on positron emission tomography.

Cardiac magnetic resonance with gadolinium contrast administration distinguishes takotsubo syndrome from acute myocardial infarction and myocarditis.^52^ Unlike these latter conditions, fibrosis depicted by late gadolinium enhancement is usually not a feature of takotsubo syndrome. Rarely, a characteristic pattern of takotsubo syndrome appears as a thin transmural band of fibrosis at the hinge points between the hyperkinetic base and dyskinetic apex or mid-cavity. This can be seen both acutely (at the time of presentation) and 4-5 months later at follow up (Figure 2). This possibly results from the opposing strong shear forces applied to the left ventricular wall. Cardiac magnetic resonance also provides a reliable assessment of right ventricular involvement, identification of left and right ventricular thrombi, and often shows the presence of a small pericardial effusion (as well as pleural effusions), particularly if the test is performed early after presentation.

Intense myocardial edema is an important feature of takotsubo syndrome (Figure 2). Edema is not only confined to regions of abnormal contractility but is present to a lesser extent within the entirety of the ventricular myocardium. Myocardial edema resolves gradually over weeks or months following the index event, typically taking much longer to recover than myocardial contractility.^53^ Reflecting this, left ventricular mass is markedly elevated and native T1 and T2 mapping values are increased during the acute phase, gradually resolving over 5-6 months during convalescence (Figure 2).^51^ Both myocardial edema and acute inflammation are detectable at presentation, but it remains unclear whether they are a consequence of takotsubo syndrome or if they represent a primary, causal inflammatory stimulus.

## Diagnostic Criteria

**Figure 3:**
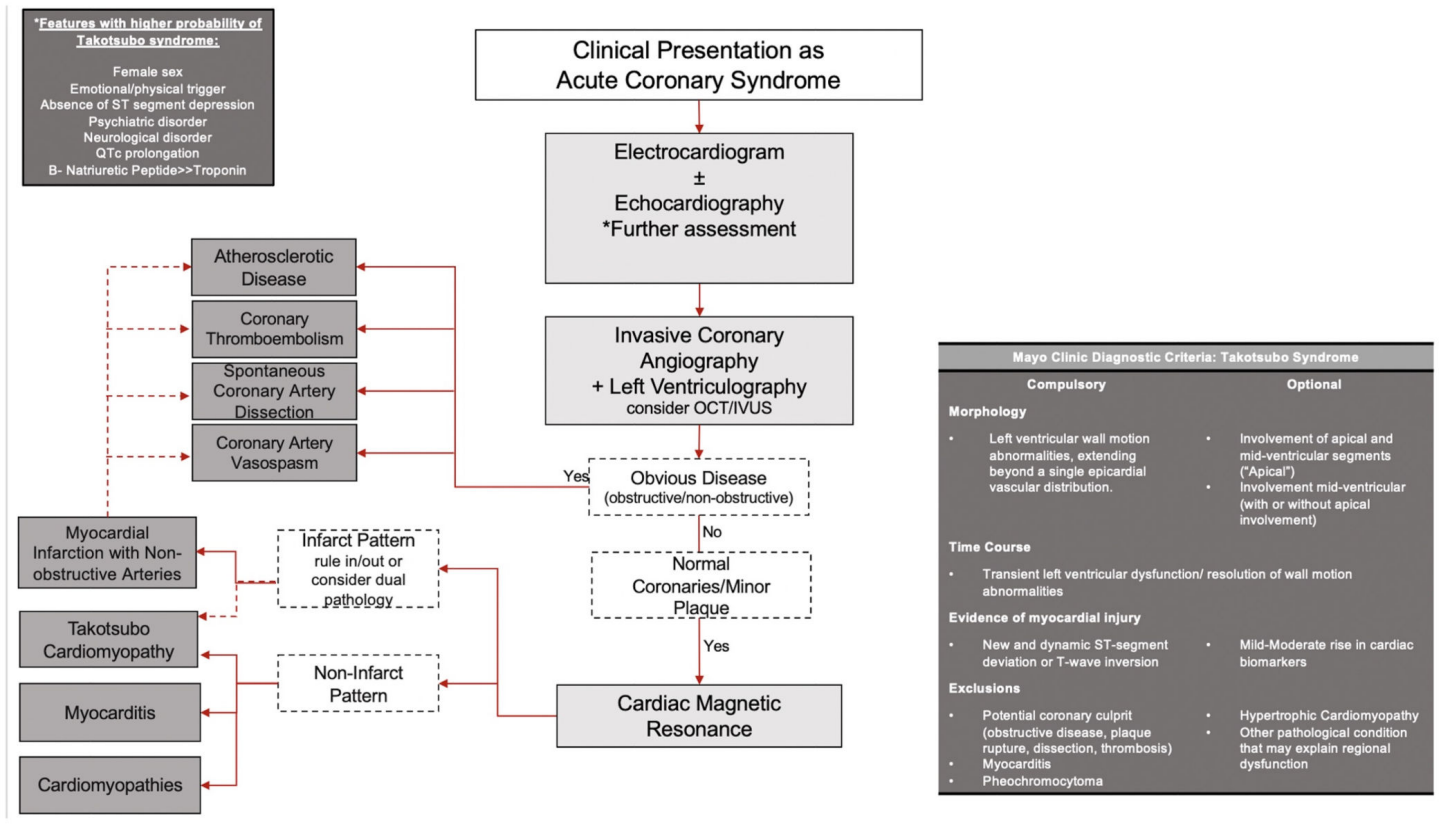
Diagnostic Criteria and Pathway for Takotsubo Syndrome OCT, optical coherence tomography, IVUS, intra-vascular ultrasound.

The diagnosis of takotsubo syndrome can be challenging because clinical features have many similarities with acute coronary syndrome and immediate early cardiac imaging is needed because of the rapid normalization of left ventricular ejection fraction. The most widely used diagnostic criteria are those proposed by the Mayo Clinic in 2004^54^ and subsequently revised in 2008^55^ (Figure 3). Other groups have proposed slightly different diagnostic conventions, such as the Gothenburg criteria^56^, Johns Hopkins criteria^57^, the Takotsubo Italian Network proposal^58^ and the Heart Failure Association Takotsubo Syndrome Taskforce of the European Society of Cardiology criteria.^59^ The underlying principle is that takotsubo syndrome is a diagnosis of exclusion since there has yet to be defined a specific diagnostic test or biomarker to identify the condition. Indeed, this extends to the post-mortem examination of fatal cases where specific diagnostic pathological features have yet to be defined, making it currently impossible to assign this diagnosis at autopsy.

Traditionally, the presence of coronary artery disease may have deterred the clinician from a diagnosis of takotsubo syndrome. Despite this uncertainty, it has become evident that takotsubo syndrome can co-exist in the presence of fixed coronary artery disease and can even be triggered by acute coronary syndrome.^15^ It is vital to look for subtle differences in clinical presentation to avoid misdiagnosis (Table 1). Equally, patients may be incorrectly diagnosed with takotsubo syndrome if coronary angiography reveals non-obstructive coronary artery disease, and the possibility of plaque rupture has not been ruled out, especially as stressful events are also associated with acute plaque rupture and type 1 myocardial infarction. The Inter-TAK diagnostic score^60^ was developed to aid the clinician in distinguishing takotsubo syndrome from acute coronary syndrome, but its performance is variable. Because understanding of the fundamental pathophysiology of takotsubo syndrome is limited, these criteria are likely to continue to evolve with time. We provide a diagnostic pathway incorporating the Inter-TAK score to aid diagnosis (Figure 3). Ultimately, the hallmark of takotsubo syndrome is the reversibility in systolic function which occurs within hours, days or weeks, in the absence of infarct-specific myocardial fibrosis.^6, 14^ The clinical implication of recovery time remains unknown and there is emerging evidence that full recovery may be slower and less complete than initially thought.

## Clinical Course

**Figure 4:**
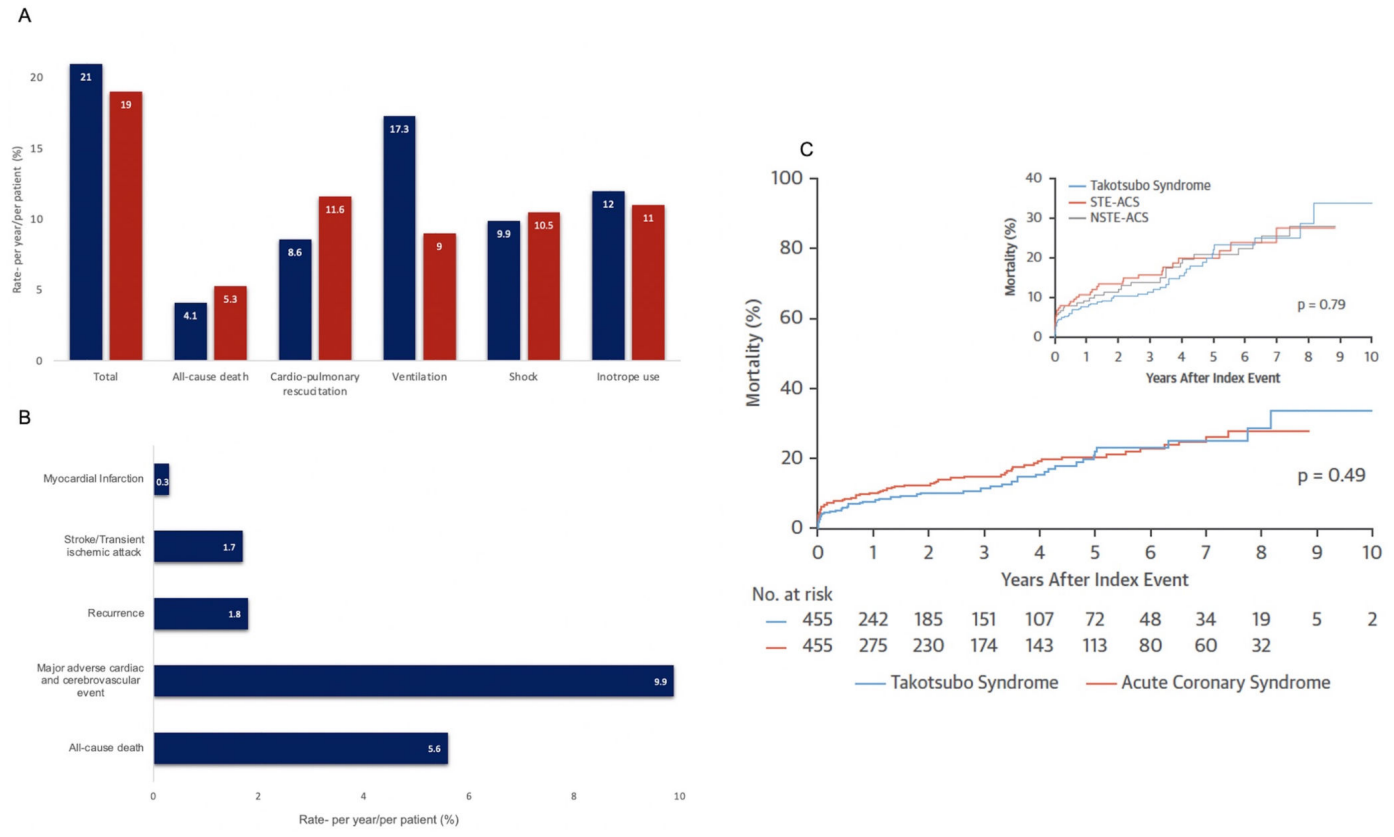
Outcomes in Takotsubo Syndrome (A), In-hospital complications in patients with takotsubo syndrome (blue) and patients with acute coronary syndrome (red). (B), long-term outcomes in patients with takotsubo cardiomyopathy. (C) Kaplan Meier curves for long-term mortality in patients with takotsubo syndrome compared with patient with acute coronary syndrome. Ghadri et al, 2018 (reference 15). STE-ACS, ST-segment elevation acute coronary syndrome, NSTE-ACS, Non-ST segment elevation acute coronary syndrome.

Despite its transient nature, patients are at substantial risk of complications during initial hospitalization period, with one in five patients at risk of serious adverse events and 1 in 25 dying.^9^ These events occur as a consequence of acute complications from the takotsubo syndrome itself (cardiogenic shock, cardiac arrest and congestive heart failure) or the critical underlying illness (respiratory failure, acute renal failure, stroke and sepsis). ^9, 10, 12, 61^ Importantly, the overall rate of in-hospital complications is comparable to that of patients with acute coronary syndrome (21% versus 19%, Figure 4).^9, 11, 13^

Fifteen to twenty percent of patients with takotsubo syndrome develop hypotension, which varies in severity.^61^ Hypotension likely reflects the underlying left ventricular systolic dysfunction, although vasodilatation, left ventricular outflow tract obstruction and mitral regurgitation may contribute. However, the extent and severity of left ventricular systolic dysfunction does not appear to correlate with systolic blood pressure or clinical heart failure. Patients with apical takotsubo syndrome are more likely to sustain a greater degree of left ventricular dysfunction than those with atypical forms. Ten percent of patients with takotsubo syndrome will develop cardiogenic shock, although these rates are surprisingly low given the magnitude and extent of left ventricular systolic dysfunction.^61^ Patients who sustain an anterior ST-segment elevation myocardial infarction and a similar degree of left ventricular systolic dysfunction are often more compromised.

Left ventricular outflow tract obstruction occurs in 10-15% of patients and may exacerbate left ventricular apical ballooning and dysfunction as it exposes the apex to higher wall stress compared to the basal myocardium.^59^ Similarly, mitral regurgitation occurs in 10-20% of patients^61^ due to tethering and systolic anterior motion of the mitral valve leaflet. These abnormalities resolve with the subsequent associated improvements in left ventricular contractility.

Atrial and ventricular arrhythmias are frequently seen, with atrial fibrillation being the commonest (3%). Life-threatening arrhythmias, such as torsade de pointes and polymorphic ventricular tachycardia, occasionally occur. ^9, 13^

Systemic thromboembolism is an important complication of takotsubo syndrome, which is often under appreciated. Rates of ischemic stroke are higher than those seen following an acute coronary syndrome and occur in 1-2% of acute hospitalizations.^9, 13, 62^ An elevated stroke risk appears to extend beyond the acute episode and is much more prominent than subsequent rates of myocardial infarction which are low (1.7 % versus 0.3%, Figure 4).^47^ The most likely embolic origin is left ventricular mural thrombus due to blood stasis within the akinetic ventricular segments (Figure 2). The spontaneous improvement in left ventricular myocardial function may initiate embolization and consequent stroke. The German Italian Stress Syndrome (GEIST) registry demonstrated that presence of left ventricular thrombi was associated with embolic cerebrovascular events in 17% of patients.^49^

Several studies^8–11, 13^ have demonstrated that long-term mortality is similar to that of acute coronary syndrome: all cause death in 5.6% of patients per year (Figure 4).^9^ Cardiovascular deaths are the commonest cause of death in both takotsubo and acute coronary syndromes.^8^ More recently, Redfors et al^13^ demonstrated that long-term mortality for takotsubo syndrome is comparable to patients with non-ST-segment elevation myocardial infarction, but lower than those presenting with ST-segment elevation myocardial infarction. Interestingly, age <50 years, male sex, physical trigger, left ventricular ejection fraction <45%, presence of atrial fibrillation, cardiac troponin concentration over 10-fold the upper limit of normal and acute neurological disease were independent predictors of 1-year mortality^8–10, 12^ but not the location (apical, basal, midventricular, focal) of myocardial ballooning.^32^

## In-Hospital Management

With no robust or clinical trial evidence available for the treatment of this condition, extrapolation of therapies known to be effective after myocardial infarction, such as beta-blocker and angiotensin-converting enzyme inhibitor therapies, have been adopted by some clinicians. In the acute setting, the focus of management relates to supportive care and treatment of complications and are predominantly based on clinical experience and expert consensus (Table 2).^25, 63^

### Heart failure

In the unusual situation where patients present with pulmonary edema, they should be treated with intravenous diuretic and nitrate therapies. Patients who sustain left ventricular outflow tract obstruction may benefit from sequential small doses of short acting beta-blocker therapy^64^, as well as small boluses of intravenous fluid, under careful monitoring in a high-dependency care environment.

The management of shock is challenging in both theory and practice. Most inotropic agents can potentially aggravate systolic dysfunction because excess catecholamines may be involved in the underlying etiology and pathophysiology of the condition. As such, expert consensus recommends mechanical approaches to provide hemodynamic support, such as intra-aortic balloon counterpulsation, temporary left ventricular assist devices and extracorporeal membrane oxygenation. ^25, 63^ However, identification of left ventricular outflow obstruction is important because hemodynamic status may be worsened by intra-aortic balloon counterpulsation in such cases. When mechanical support is not available, low-dose levosimendan is licensed in some countries as a catecholamine-sparing positive inotrope (Table 2).^65,66^

### Arrhythmias

The management of ventricular arrhythmias is dependent on the clinical picture and mirrors the general principles of acute arrhythmia management. It is important to avoid QT segment prolonging medications as these may worsen the likelihood of developing ventricular arrhythmias due to the high risk of further QTc prolongation. In clinical practice, electrical pacing of torsade de pointes have proven successful to bridge the patient and allow recovery from the acute phase. Although uncommon, if high degree atrioventricular block is present, inotropes and permanent devices should be avoided, semi-permanent (active fixation lead connected to an externalized pulse generator) or temporary right ventricular pacing may be considered if there is hemodynamic instability.^25, 67^ Patients who present with life-threatening ventricular arrhythmias and takotsubo syndrome should be considered for an implantable cardiac defibrillator. However, there is no specific evidence base to support this approach and there are no long-term outcome data of device discharge rates in patients with takotsubo syndrome.^25^

### Thromboembolism

Patients who have a confirmed left ventricular thrombus should be anticoagulated for at least 3 months or until resolution. Although no guidelines currently exist for the treatment of takotsubo syndrome, the relatively high rates of systemic thromboembolism would suggest better identification of thrombus on cardiac imaging is needed prior to discharge and should be considered in high-risk patients, such as those with severe left ventricular dysfunction (ejection fraction <30%) and apical ballooning.

## Long-Term Management

As with in-hospital management, no therapeutic interventions have been shown to reduce recurrences or any other major cardiovascular events in patients with takotsubo syndrome. The focus is therefore to ensure the treatment of associated risk factors and concomitant disease.

### Concomitant Disease

Patients with concomitant coronary artery disease (bystander disease) should continue to receive preventative therapies including antiplatelet, statin and angiotensin-converting enzyme inhibitor therapies. Furthermore, those who have dual pathology (concurrent acute myocardial infarction) may require dual antiplatelet therapy and treatment should be tailored to individual patients [Daghem et al, A tear and a broken heart.2022]. Where patients demonstrate a degree of persistent left ventricular systolic dysfunction, they should be managed and treated for left ventricular systolic dysfunction according to established guidelines.^63^ However, in the vast majority of patients who have normalization of their left ventricular ejection fraction, there is no clear evidence that beta blocker therapy improves long-term survival benefit or reduces the recurrence of takotsubo syndrome.^9, 65, 68^ Similarly, the evidence for angiotensin-converting enzyme inhibitor or angiotensin receptor blocker therapy is contradictory and uncertain.^9, 65, 69^ Randomized controlled trial evidence is urgently needed. In the meantime, each clinician should continue to apply their best judgement but conservative medical management appears to be the most appropriate approach in the absence of treatable risk factors or co-morbidities.

### Risk factors

Psychiatric disorders, usually anxiety or depression, are common in patients with takotsubo syndrome, suggesting that some patients might benefit from a combined psycho-cardiologic rehabilitation. Cognitive-behavioral therapy alongside cardiac rehabilitation can improve mental health and reduce negative thinking compared to cardiac rehabilitation alone.^70^ Studies are currently underway to establish whether structured exercise training and mental well-being programs can improve cardiac energetics and attenuate cardiac limitation on exercise after takotsubo syndrome (PLEASE study, NCT04425785). Whether anti-depressant or other psychiatric therapies might provide clinical benefit in such patients is controversial and has not been investigated.

## Etiology And Pathophysiology

**Figure 5:**
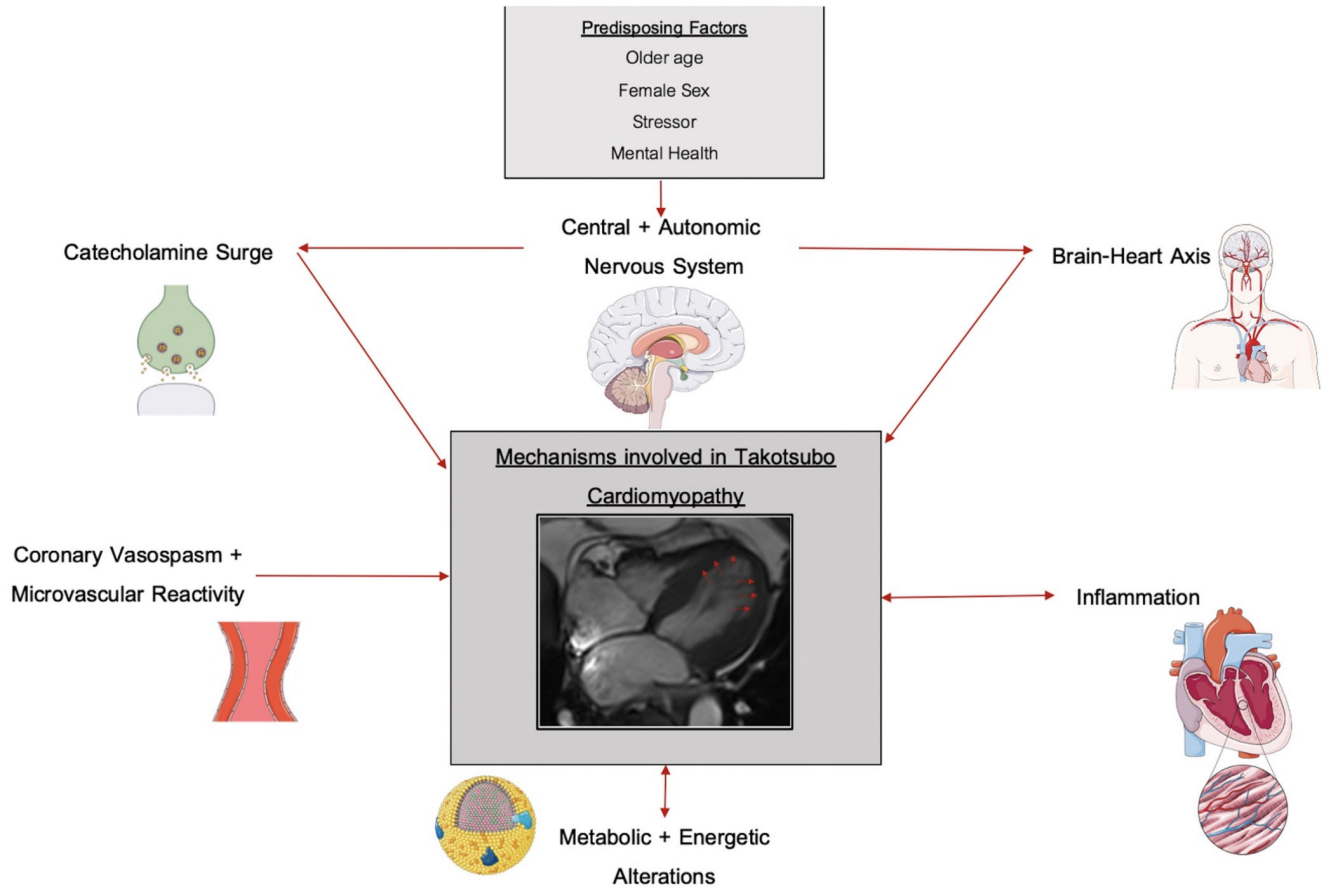
Mechanisms Involved in Takotsubo Syndrome

Understanding the underlying etiology and pathophysiology (Figure 5) is critical if appropriate treatments are to be developed for the acute episode as well as the prevention of subsequent long-term recurrent events. Furthermore, it is unknown whether the underlying mechanism varies according to the anatomical variation of the left myocardial ballooning.

### The Adrenergic Hypothesis

Endogenous adrenergic surge is the most established theory for the pathogenesis of takotsubo syndrome and is intuitive given the strong association with sudden unexpected stress and major physical illness or trauma.

Two principal aspects to this mechanism need to be considered: the release of catecholamines and the cardiac response to catecholamines. The locus coeruleus, located in the posterior part of the rostral pons is the primary origin of norepinephrine in the brain. Receiving multiple inputs from the limbic system, it regulates the homeostatic response to emotions.^71^ The locus coeruleus is triggered by emotional stimuli and leads to the production of norepinephrine, which in turn activates the hypothalamic-pituitary-adrenal axis.^71^ In response to this activation, the adrenal medulla releases epinephrine and norepinephrine into the circulation, thereby increasing plasma catecholamine concentrations.

Iga and colleagues were the first to describe takotsubo syndrome in a patient with pheochromocytoma.^72^ This particular case report was important as it first established the relationship between takotsubo syndrome and elevated catecholamine concentrations. Subsequent observational studies have demonstrated elevated blood catecholamine concentrations in the acute phase, although this has not been a universal finding.^73^ Preclinical studies have shown that intravenous or intraperitoneal administration of norepinephrine, epinephrine or isoprenaline can reproduce the characteristic reversible apical left ventricular ballooning coupled with basal hypercontractility.^74, 75^

Independent of a systemic increase in catecholamine concentrations via the hypothalamic-pituitary-adrenal axis, a local neurally mediated increase in catecholamine release at myocardial level may also occur.^76^ Apart from the locus coeruleus, neural impulses descend (from the rostral pons) into posterior hypothalamus triggering norepinephrine release from sympathetic nerve terminals supplying the myocardium and coronary circulation. Increase in local production may explain why plasma catecholamine concentrations are not always elevated and several studies have demonstrated myocardial sympathetic hyperactivity.

The second aspect is the cardiovascular response to a surge in catecholamines and how this results in left ventricular dysfunction. Mammalian hearts demonstrate the highest density of beta-adrenergic receptors in the apex although the distribution of beta-1 and beta-2 adrenoreceptors has never been mapped in the human heart.^23^ Increased responsiveness of the left ventricular apex to catecholamines could explain the characteristic apical ballooning of takotsubo syndrome.^77^

Some have suggested direct myocardial injury from catecholamine excess. Furthermore, catecholamines released directly into the myocardium *via* sympathetic nerves may have a greater ‘toxic’ effect than that reaching the heart via the bloodstream.^77^ Indeed, norepinephrine spillover can decrease myocyte viability, resulting in contraction band necrosis, which is one of the histological findings reported in takotsubo syndrome (Figure 2). ^78, 79^ Contraction band necrosis is also found in patients with pheochromocytoma and subarachnoid hemorrhage, which have also been attributed to catecholamine excess.^78, 79^

**Figure 6:**
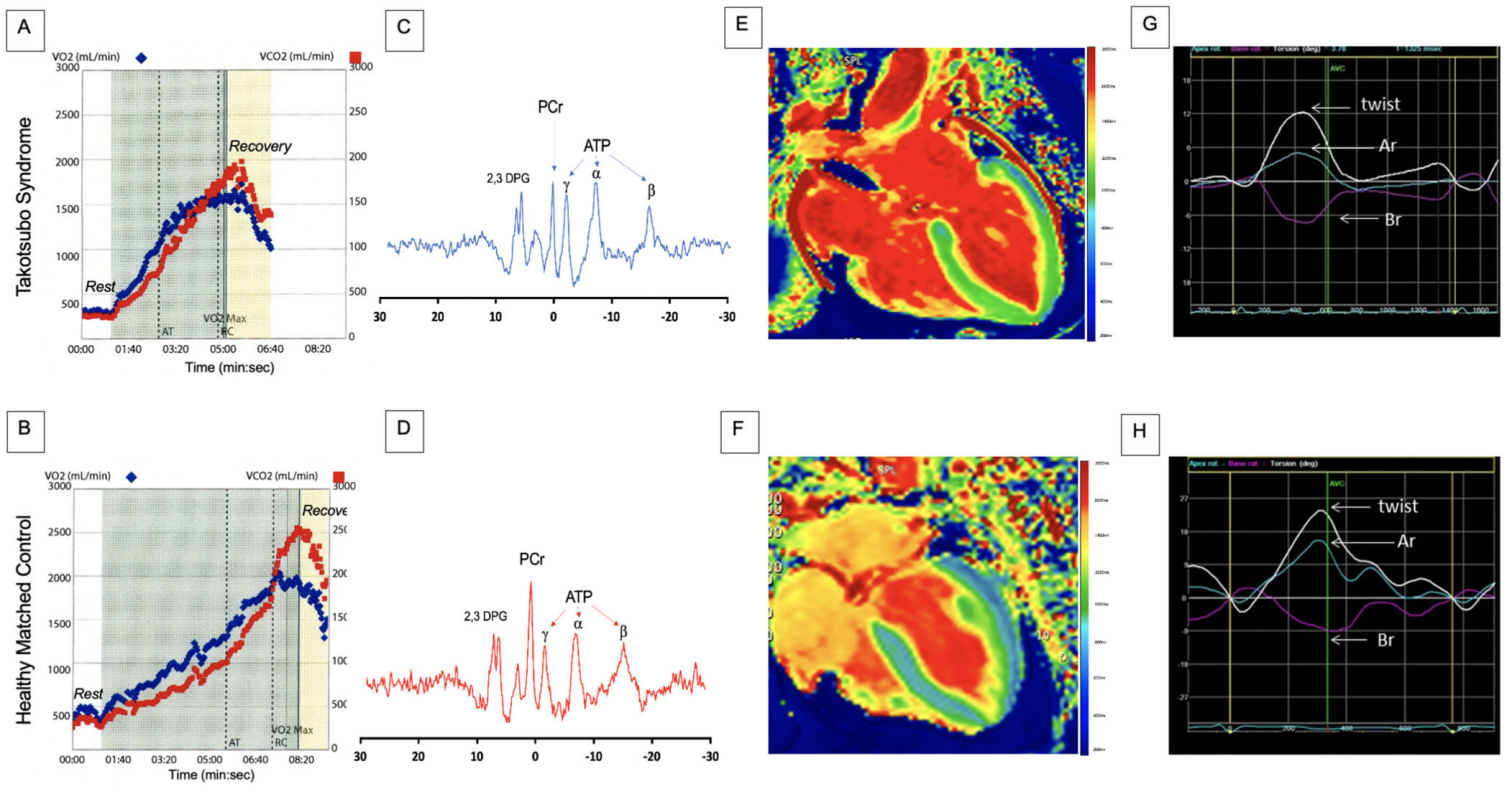
Abnormal Exercise Capacity, Energetics and Cardiac Performance in Patients with Takotsubo Syndrome Cardiopulmonary exercise (treadmill) data from (A) a patient with prior takotsubo syndrome 20 months previously and (B) an age- and sex-matched healthy control subject. The maximal oxygen consumption (VO_2_, blue dots) achieved by the patient with takotsubo syndrome is markedly reduced, with an earlier anerobic threshold and a shorter duration of exercise when compared to the healthy control subject. ^31^Phosporus magnetic resonance spectrum acquisition for a patient with acute takotsubo syndrome (C) and healthy volunteer (D) showing reduced phosphocreatine/adenosine triphosphate ratio. Resonances corresponding to phosphocreatine (PCr), γ, ß, and α adenosine triphosphate (ATP), and 2,3-diphosphoglycerate (2,3 DPG). Manganese-enhanced magnetic resonance imaging in (E) a patient with acute takotsubo syndrome and (F) an age and sex-matched volunteer. The patient demonstrates abnormal calcium activity (green) throughout the mid ventricle and apex with preserved calcium activity in the basal segments (blue). In comparison, the healthy control subject demonstrates normal calcium activity throughout the myocardium (blue). Twist curves in (G) a patient with takotsubo syndrome at 2-year follow up and (H) an age and sex-matched healthy control volunteer. The healthy subject demonstrates the characteristic early systolic twist in a clockwise rotation at the apex (blue trace) and in counter clockwise rotation at the base (purple trace), occurring during isovolumic contraction. This is followed by counter clockwise apical rotation (Ar; blue) and clockwise basal rotation (Br; purple), which results in the net systolic twist during left ventricular ejection (twist, white line). In comparison, the patient with takotsubo syndrome demonstrates incomplete recovery of left ventricular twist, predominantly due to the reduced apical rotation. VO_2_ (mL/min), volume of oxygen inspired, VCO_2_ (mL/min), volume of carbon dioxide exhaled, AT, anerobic threshold, t(s), time, VO_2_ Max, maximal volume of oxygen inspired.

Some authors have hypothesized that a surge in adrenoreceptor stimulation dysregulates myocardial calcium-handling. Immunohistochemistry studies suggest that calcium-regulating proteins such as phospholamban, sarcoendoplasmic reticulum calcium-ATPase (SERCA) and sarcolipin are altered, resulting in depressed left ventricular contraction during acute takotsubo syndrome.^80^ Studies are currently underway that aim to assess myocardial calcium-handling in patients with takotsubo syndrome (MEMORY study, NCT04623788, Figure 6). Others have suggested that adrenoreceptor stimulation can create an imbalance of oxygen supply and demand, thus creating myocellular hypoxia. Hypoxia is further exacerbated by metabolic changes and electrolyte imbalances from alterations in membrane permeability that may contribute to myocardial toxicity.

At high concentrations, epinephrine can act as a negative inotrope via ligand-mediated stimulatory Gs protein. Following cyclic adenosine monophosphate-dependent phosphorylation of the β2-adrenoreceptor, receptor coupling is switched from the Gs to Gi protein.^81^ The Gi protein prevents excessive activation of the myocyte and reduces contractility, which protects the myocardium from the effects of excess catecholamine stimulation and restricts cardiac damage.^81^ The switch back to Gs protein is responsible for the rapid recovery in these patients and could explain resolution of left ventricular dysfunction. However, this theory does not explain all of the features of takotsubo syndrome, such as the presence of marked myocardial edema, absence of a stressor in some patients, and why only two-third of patients have elevated catecholamine concentrations.

In the final analysis, it should be remembered that patients with takotsubo syndrome are neither tachycardic nor hypertensive at presentation, and this contrasts with all other conditions known to have high catecholamine surges. Thus, takotsubo syndrome might affect the autonomic nervous system in a much more complex way than a simple and pure catecholamine surge.

### Brain-Heart Axis

For many patients, psychological stress is the central trigger for takotsubo syndrome, even in the presence of a physical illness, which arguably induces an element of psychological stress. Patients with takotsubo syndrome are more likely to have pre-existing psychiatric illness. Patients with depression and anxiety have up regulated micro-ribonucleic acid (miRNA) 16 and 26a, and in a rodent model of miRNA 16 and miRNA 26a overexpression, exogenous epinephrine was associated with apical wall motion abnormalities.^82, 83^ This mechanism is consistent with a predilection of the myocardium to develop takotsubo syndrome in response to stress and could explain the high prevalence of pre-existing and acute psychiatric illness in affected individuals.

‘Neurocardiogenic stunning’ of the heart is a well-recognized complication following acute neurological injury, and 20-30% of patients develop transient left ventricular systolic dysfunction, highlighting the complex brain-heart interaction.^84^ Patients with takotsubo syndrome demonstrate altered neuronal connectivity in several stress associated limbic regions. Altered neuronal activity is predominately seen in the hippocampus, amygdala, cingulate gyrus and insula and are important in regulating emotional responses and the autonomic nervous system.^85^ Furthermore, ^18^F-fluorodeoxyglucose positron emission tomography imaging has demonstrated heightened amygdala activity years before patients develop takotsubo syndrome.^85^ This suggests that when presented with a potential trigger, such individuals may have a weakened ability to respond appropriately, and an imbalance of the sympathetic and parasympathetic nervous systems results in myocardial injury similar to that of an acute neurological insult.^86^

Several case reports have described takotsubo syndrome in patients following cardiac transplantation. Given denervation of the heart following cardiac transplantation, this does argue against a direct neural stimulus as the trigger for takotsubo syndrome and suggests that humoral mechanisms may have a more prominent role. Indeed, it has been postulated that parasympathetic denervation may lead to upregulation of catecholamines and beta-adrenoreceptors.^87^ Combined with impaired neural innervation, this may result in an exaggerated response to catecholamines and susceptibility to takotsubo syndrome. The brain-heart axis is yet to be properly explored and possibly holds the answers to a number of questions that elude us with regards to takotsubo syndrome and possibly other cardiac diseases.

### Coronary vasospasm and microvascular reactivity

Initial cases from Japan demonstrated multivessel epicardial coronary vasospasm on coronary angiography, raising the possibility that multivessel vasospasm may be a causative factor in the pathogenesis of takotsubo syndrome.^77^ However, repeated provocation testing in patients with takotsubo syndrome found such responses to be inconsistent with only approximately 20% of patients demonstrating reproducible vasospasm.^88^ Furthermore, such provocation testing is not physiological, has poor reproducibility and is of uncertain relevance to spontaneous epicardial spasm.

Microvascular dysfunction and impaired reactivity does appear to be a feature of takotsubo syndrome with demonstrable reversible abnormalities in both the coronary flow reserve and the index of microvascular resistance. Post-menopausal women have age-related and estrogen deficiency-related coronary vasomotor dysfunction. Under physiological circumstances, estrogen improves coronary blood flow via endothelium-dependent and -independent mechanisms, but its deficiency results in increased sympathetic drive and endothelial dysfunction.^89^ These changes may in part explain the preponderance of takotsubo syndrome to occur in post-menopausal women.

Nuclear myocardial perfusion studies have reported reduced apical perfusion, which gradually recovers at 1 and 6 months.^90^ Such microvascular dysfunction and abnormal perfusion may be driven by vasoconstrictor mediators such as endothelin, catecholamines and the associated reactive oxygen species.^91^ Another possible explanation may be myocardial inflammation leading to direct myocyte injury including vascular endothelial injury causing shedding of the endothelial glycocalyx and consequent myocardial edema. However, there are issues of cause and effect for both myocardial edema and microvascular dysfunction, which may be a consequence rather than a cause, of the acute episode.

Wittstein^92^ postulated the interaction between sympathetic overactivity and microvascular dysfunction and its impact on clinical presentation in patients with takotsubo syndrome. High-risk individuals with elevated sympathetic tone and vasomotor dysfunction (post-menopausal status, depression and treatment with serotonin re-uptake inhibitors) may only require a mild stimulus to precipitate microvascular ischemia and subsequent myocardial stunning.^92^ Conversely, low-risk individuals with normal sympathetic and vasomotor tone will likely require a much larger catecholamine surge to precipitate acute takotsubo syndrome. This may explain why some patients present following seemingly mild triggers.

### Metabolic and energetic alterations

Current data demonstrate metabolic and energetic impairment in acute takotsubo syndrome followed by a protracted and incomplete recovery.^43^ Preclinical studies have shown increased myocardial glucose uptake, and although there were appropriate increases in enzymes involved in the glycolytic pathway, there was a reduction in the available metabolites of glycolysis.^91^ Thus resulting in a decreased production of Kreb’s cycle intermediates and adenosine triphosphate. It is unknown whether this decrease is due to a state of myocardial metabolic enhancement leading to exhaustion and loss of metabolites or if this is due to myocardial metabolic stunning leading to enzymatic blockade of the glycolytic, beta-oxidative or pentose phosphate pathways.^91^

The *in vivo* gold standard for exploring myocardial energetics is 31-phosphorus cardiac magnetic resonance spectroscopy. Resting cardiac energetic status (phosphocreatine to γ-adenosine triphosphate ratio) is reduced in patients with acute takotsubo syndrome (Figure 6). Whilst there is some recovery by 4 months of followup, it has still not completely normalized.^93^ Indeed, abnormal long-term myocardial metabolism may explain why patients continue to be symptomatic and have recurrent events despite apparent recovery of left ventricular ejection fraction.^51^

### Inflammatory Mechanisms

There is growing evidence to support the presence of myocardial inflammation in the acute phase of takotsubo syndrome. Although this will, in part, be a reaction to the precipitating event, it may be both cause and effect. Furthermore, a maladaptive persistent subacute or chronic inflammation may contribute to long term cardiac dysfunction.

In a multicenter study (TERRIFIC, NCT02897739)^43^, patients with takotsubo syndrome had greater retention of ultra-small superparamagnetic particles of iron oxide in both ballooning and non-ballooning left ventricular segments during the acute phase. As ultra-small superparamagnetic particles of iron oxide are predominantly phagocytosed by activated tissue-resident macrophages, the main cellular protagonists of the myocardial cellular inflammation in acute takotsubo syndrome appear to be macrophages, whereas acute myocarditis is principally lymphocyte-mediated. Additionally, serum interleukin-6 and chemokine (C-X-C motif) ligand 1 concentrations as well as classic CD14^++^CD16 monocytes are increased, whereas intermediate CD14^++^CD16^+^ and non-classic monocytes are reduced in patients with takotsubo syndrome. At 5 months of follow-up, enhancement with ultra-small superparamagnetic particles of iron oxide was no longer detectable in the myocardium, although persistent elevations in serum interleukin-6 concentrations and reductions in intermediate CD14^++^CD16^+^ monocytes were present. Therefore, takotsubo syndrome is characterized by a myocardial macrophage inflammatory infiltrate, changes in the distribution of monocyte subsets, and an increase in systemic proinflammatory cytokines. Many of these changes persisted for at least 5 months, suggesting a low-grade chronic inflammatory state. Furthermore, post-mortem examination of human hearts from patients who died during the acute phase of the condition demonstrated that these macrophages are predominantly of the M1 pro-inflammatory type as opposed to the reparative M2 type.^94^ The presence of M1 macrophages and the persistence of the intermediate (CD14^++^CD16^+^) monocyte subset at 5 months follow-up are strongly indicative of a less reparative and more pro-inflammatory state compared to similar stages of patients with acute myocardial infarction. It remains unclear, however, if this inflammatory activation is causative or consequential to takotsubo syndrome. Irrespective of this, these findings offer explanation for the low-grade chronic inflammatory substrate with subsequent evolution of acute takotsubo into long term heart failure.

### Persistent or Pre-existing Syndrome

The heart failure phenotype of takotsubo syndrome has been thoroughly characterized in a cohort of predominantly symptomatic patients (the HEROIC study, NCT02989454)^51^ and demonstrates preserved ejection fraction, impaired cardiac energetic status, cardiac limitation on exercise (reduced peak VO_2_ and increased VE/VCO_2_ slope during cardiopulmonary exercise testing), reduced apical myocardial anti-clockwise rotation during systole with altered torsion and twist, and possibly microscopic fibrosis (Figure 6).^43, 51^ Similarly, cardiac biomarkers, such as B-type natriuretic peptide, remain mildly elevated long-term.^95^ This persistence of long-term myocardial abnormalities does beg the question of whether such abnormalities pre-date the index takotsubo syndrome. This would be consistent with the predisposition for recurrent takotsubo events. Of course, it is difficult to know whether the myocardium in these patients was ‘healthy’ to begin with or there was a pre-existing subtle and undiagnosed cardiomyopathy that is brought to light during an acute stressful event. If this were the case, then it would imply that their cardiac function may never return to ‘normal’ and has in fact returned to baseline.

## Future Directions

With advances in imaging, patients being investigated for takotsubo syndrome should undergo multimodal non-invasive imaging. Not only does this increase the accuracy of the diagnosis, but it also allows for risk stratification and prognostication. The precise diagnosis of takotsubo syndrome does rely on the combination of clinical context, echocardiography, cardiac catheterization and cardiac magnetic resonance imaging. Future exploration of a single sensitive and specific diagnostic test would greatly simplify clinical care pathways and would give added impetus to future therapeutic trials by identifying a more homogeneous patient population.

There is a major lack of evidence to guide management in patients with takotsubo syndrome and this needs to be the focus of future research. There are two main therapeutic domains that need addressing. First, the management of acute takotsubo syndrome needs to be defined, especially guidance on the treatment of severe complications, such as cardiogenic shock. Second, the prevention of recurrent major adverse cardiac and cerebrovascular events is crucial. This includes the role of heart failure therapies to prevent re-occurrence of takotsubo syndrome, anticoagulant therapies to prevent ischemic strokes and psychiatric interventions, specifically in those with underlying mental health problems. Randomized controlled trials such as the n-acetyl cysteine and ramipril takotsubo syndrome trial (NACRAM, ACTRN12616000781448)^96^ and optimized pharmacological treatment for broken heart syndrome (BROKEN-SWEDEHEART, NCT04666454) are currently assessing potential interventions and will be invaluable in informing treatment guidelines.

Given the presence of substantial and protracted myocardial inflammation, a potential role for anti-inflammatory therapies may have a role in the recovery from takotsubo syndrome. Long-term cardiac energetic impairment may also be one reason why patients continue to have symptoms and are prone to recurrent episodes despite apparent recovery of left ventricular ejection fraction. Interventions targeting these impairments of cardiac metabolism could be beneficial. For example, the benefits of sodium-glucose transporter 2 inhibitor therapy in heart failure may be related to an improvement in cardiac efficiency due to a shift in cardiac metabolism and could potentially be applicable to the takotsubo syndrome.^97, 98^ Further studies of specific substrate utilization and enhancement of cardiomyocyte metabolic pathways is an attractive investigative pathway.

Patients with takotsubo syndrome have persistent symptomatic and functional impairment as well as recurrent major events and it is essential that future studies focus on the long-term assessment of cardiac function, metabolism, symptoms and clinical outcomes. This will be the focus of the Inter-TAK registry (NCT01947621),^11, 47^ which has launched a 10-year follow up study in patients with takotsubo syndrome. This will also help identify different subtypes of takotsubo syndrome that may require distinct cause-specific interventions.

## Conclusions

Takotsubo syndrome is increasing in incidence and becoming more widely recognized in the medical community. The diagnosis can be challenging, and clinicians must look for signs distinguishing takotsubo syndrome from other causes of acute myocardial injury. The use of multi-modality imaging may help in this regard and improve our understanding of its underlying etiology and pathophysiology. Currently takotsubo syndrome remains a poorly understood condition with substantial morbidity and mortality without proven or effective treatments that urgently needs to be addressed.

## Figures and Tables

**Table 1:** Differential Diagnosis in Takotsubo Syndrome

	Takotsubo syndrome	Myocardial Infarction with Non-obstructive Coronary Arteries	Type 2 Myocardial Infarction	Type 1 Myocardial Infarction
**Characteristic Clinical Features**	Acute chest pain or dyspnea Preceding stressor* Can be associated in the context of other illness Pre-existing psychiatric illness	Acute chest pain Varies depending on underlying pathology Requires clinical evidence of myocardial infarction	Acute chest pain Supply or demand imbalance Tachycardia or hypotension Concomitant illness Usually have co-morbidities	Acute chest pain Traditional cardiovascular risk factors
**Investigations Electrocardiogram**	Widespread ST-segment or T wave abnormality QTc prolongation (late)	New Ischaemic changes	New ischaemic changes Tachycardia	Territorial ST-segment or T wave abnormality
**Bloods**	Moderate cardiac troponin elevation Marked BNP and CRP elevation	Elevated troponin >99^th^ centile	Elevated troponin >99^th^ centile	Marked cardiac troponin elevation Moderate BNP and CRP elevation
**Echocardiography**	Temporary left ventricular dysfunction regional wall motion abnormality depending on subtype of takotsubo (apical, basal, focal)	Normal or persistent left ventricular impairment regional wall motion abnormality	Normal or persistent left ventricular impairment May have regional wall motion abnormality	Normal or persistent left ventricular impairment regional wall motion abnormality
**Coronary Angiography**	Normal or non-obstructive coronary artery disease Apical ballooning on left ventriculogram	Normal or nonobstructive coronary artery disease Evidence of acute plaque rupture on intra-vascular ultrasound or optical coherence tomography	Normal, nonobstructive or obstructive coronary artery disease	Obstructive disease
**Magnetic Resonance Imaging**	No persistent late gadolinium enhancement Elevated Native T1/T2	Late gadolinium enhancement regional wall motion abnormality	Normal or late gadolinium enhancement May have regional wall motion abnormality	Late gadolinium enhancement-infarct pattern Elevated native T1/T2 regional wall motion abnormality

BNP, B-type natriuretic peptide, CRP, C-reactive protein

* Not always

**Table 2:** Management Pathway in Patients with Takotsubo Syndrome

Management	
**Acute Complications**
Heart Failure/Pulmonary Oedema	Diuretics, Nitroglycerin (if no left ventricular outflow obstruction)
Left Ventricular Outflow Obstruction	Consider: IV fluids, Beta blocker (if no heart failure), left ventricular assist device * Avoid: diuretics, nitroglycerin, intra-aortic balloon pump
Shock/Pump Failure	Consider: left ventricular assist device, VA-ECMO * Avoid: inotropes such as epinephrine, norepinephrine, dobutamine, milrinone, isoprenaline
Arrythmias	*Ventricular arrythmias:* Beta blocker, Magnesium, Direct current cardioversion *Avoid: QT prolonging drugs *High degree A V block:* Temporary pacing *Avoid: Beta blockers, permanent devices
Thromboembolism	*LV Thrombus:* Anticoagulation for at least 3 months *Consider: Prophylactic anticoagulation in patients with left ventricular ejection fraction <30% and apical ballooning
**Pre-Discharge**	
Bystander Coronary Disease	Secondary Prevention: antiplatelet and statin therapy
Left Ventricular Dysfunction	Heart Failure treatment: Beta blockers, Angiotensin converting enzyme inhibitors or Angiotensin receptor blockers, Diuretics
Tachycardia	Beta blocker, Ivabradine (if in sinus and cannot tolerate beta blockers)
Assess for Left Ventricular Thrombus	Cardiac imaging and assessment for anticoagulation
**Long- Term**	
Recurrence Prevention	Continue Angiotensin converting enzyme inhibitors or Angiotensin receptor blocker therapy
Address Risk Factors	Underlying Mental health conditions

VA-ECMO, venous to arterial extra-corporeal membrane oxygenation

## Non-Standard Abbreviations And Acronyms

BNP: B-type natriuretic peptide
NT-proBNP: N-terminal inactive molecule
miRNA: micro-ribonucleic acid
SERCA: sarcoendoplasmic reticulum calcium-ATPase
